# Use of Instructional Therapeutic Play during the preoperative nursing consultation of a child with orofacial clefts

**DOI:** 10.1590/1980-220X-REEUSP-2026-0112en

**Published:** 2026-10-12

**Authors:** Mariana Martire Mori, Camila Moraes Garollo Piran, Alana Vitória Escritori Cargnin, Laís Moreira Martins, Maricy Morbin Torres, Marcela Demitto Furtado

**Affiliations:** 1 Universidade Estadual de Maringá, Programa de Pós-Graduação em Enfermagem, Maringá, PR, Brazil.; 2 Universidade Estadual de Maringá, Departamento de Enfermagem, Maringá, PR, Brazil.

**Keywords:** Play and Playthings, Preoperative Care, Pediatric Nursing, Cleft Palate, Nursing Process

## Abstract

**Objective:**

To report the use of Instructional Therapeutic Play during the preoperative nursing consultation of a child with an orofacial cleft.

**Method:**

A case study conducted at an association that supports children with orofacial clefts located in northwestern Paraná, Brazil. Data were collected in May 2025 during a nursing consultation using Instructional Therapeutic Play, book reading, and drawing activities to prepare the child for surgery. The consultation was audio-and video-recorded with consent, and the data were analyzed descriptively. The study was approved by the Research Ethics Committee under Opinion 7,041,250/2024.

**Results:**

The study included an 8-year-old girl scheduled to undergo cleft palate repair surgery. She presented fear and anxiety related to the surgical procedure, particularly fear of not waking up after anesthesia. The use of Instructional Therapeutic Play enabled the child to understand the procedure, express her concerns, and, at the end of the session, she reported feeling calmer.

**Conclusion:**

Instructional Therapeutic Play promoted emotional expression and facilitated the surgical preparation of the child with an orofacial cleft.

## INTRODUCTION

Orofacial clefts are congenital malformations affecting the facial region that result from failure of the lip and/or the primary and secondary palate to develop during the early stages of embryonic development. These anomalies may affect, to varying degrees, the lip, oral cavity, and nasal cavity. According to their extent, they are classified into three major groups: cleft palate, cleft lip, and cleft lip and palate. They may occur as isolated defects, accounting for approximately 70% of cases, or be associated with a wide variety of genetic syndromes, representing the remaining 30%^(1)^.

In Brazil, the estimated incidence of orofacial clefts is approximately one case per 650 live births, corresponding to a rate of 1.53 per 1,000 live births. Other attempts to estimate this prevalence have been limited by methodological factors, such as the use of small samples and reliance on local hospital records, compromising the reliability and generalizability of the findings^(2)^.

Surgical correction of the malformation should be performed during the first year of life to prevent impairment of important functions, such as speech and swallowing, thereby improving the quality of life of children affected by these anomalies. The integrated work of a multidisciplinary team is essential for the effective management of orofacial clefts, given the anatomical, functional, and psychosocial complexity associated with this condition^(3)^.

In this context, preoperative educational sessions conducted by multidisciplinary teams have been shown to provide significant benefits for children undergoing surgical procedures, including reduced hospital stay and lower analgesic use^(4)^. Therefore, it is an essential component of pediatric nursing practice to seek strategies that promote children’s active engagement in the care process and help reduce the potential trauma associated with healthcare interventions^(5)^.

One strategy to promote children’s active participation is the use of Therapeutic Play (TP), whose benefits have been extensively documented in the scientific literature, highlighting its importance for child development and well-being and supporting its systematic implementation across different pediatric healthcare settings^(6)^.

Among the different modalities, Instructional Therapeutic Play (ITP) is considered the most appropriate approach for preparing children for hospitalization and therapeutic procedures^(5)^. Evidence demonstrates that the use of TP in the care of hospitalized children contributes to reducing anxiety and fear in both children and their families, thereby promoting more humanized nursing care^(6)^.

Few studies have examined the effects of ITP during invasive procedures in children. Nevertheless, pediatric nurses play a central role in designing and conducting innovative clinical studies investigating the effects of TP on promoting child health and reducing stress-related behaviors, which may intensify potentially traumatic experiences during healthcare delivery^(7)^. Therefore, this clinical study aimed to report the use of ITP during the preoperative nursing consultation of a child with an orofacial cleft.

## METHOD

### Study Design

This is a single-case report describing the application of ITP during a nursing consultation for the surgical preparation of a child with an orofacial cleft. Case reports are an important source of information that contributes to improving healthcare and treatment, particularly when they describe care strategies that are still underused in clinical practice^(8)^.

### Setting

The study was conducted at an association located in a municipality in northwestern Paraná, Brazil, that provides free multidisciplinary care for children and adolescents with orofacial clefts. The institution currently serves approximately 500 patients from 80 municipalities across the region. The multidisciplinary team includes professionals in psychology, nutrition, speech-language pathology, dentistry, social work, and educational support. Other specialties involved in the care of children with orofacial clefts provide services at a hospital located in another municipality in eastern Paraná, where children are referred for surgical procedures.

### Selection Criteria

This case report was based on a nursing consultation in which ITP and play-based strategies were used with a child with an orofacial cleft. The patient was seen by a nurse who was also a master’s student in nursing and conducting a research project at the institution after being referred by the service psychologist, who had observed the child’s fear of undergoing surgery.

### Data Collection

Data were collected during a single consultation conducted in May 2025, one month before the scheduled surgical procedure, in an individual session held at the association. The nursing consultation was structured into two phases: consultation with the family caregiver and consultation with the child. Data collection lasted approximately 60 minutes, with 20 minutes devoted to the family caregiver and 40 minutes to the child.

Initially, the child’s mother attended an individual consultation to provide additional information regarding the family and health context. Subsequently, the child participated in an individual session that began with the complete reading of the 16-page book “*Quando eu sinto medo*”, which addresses the recognition of and coping with fear during childhood^(9)^. The child was then asked to draw a picture in response to the question, “What are you afraid of regarding the surgery?”. After completing the drawing, she was invited to explain its meaning.

As the nursing consultation progressed, the nurse conducted an ITP session as both an assessment and nursing intervention strategy to demonstrate how the surgical procedure would be performed. Various toys closely resembling real clinical materials were used, including a doll with an orofacial cleft, a nurse doll, gauze, a face mask, gloves, a peripheral intravenous catheter, a stethoscope, a syringe, a multiport extension set, Micropore tape, a tourniquet, an intravenous infusion set, surgical incision adhesive strips, a toy spoon, plate, and cup, as shown in Figure 1.

**Figure 1:**
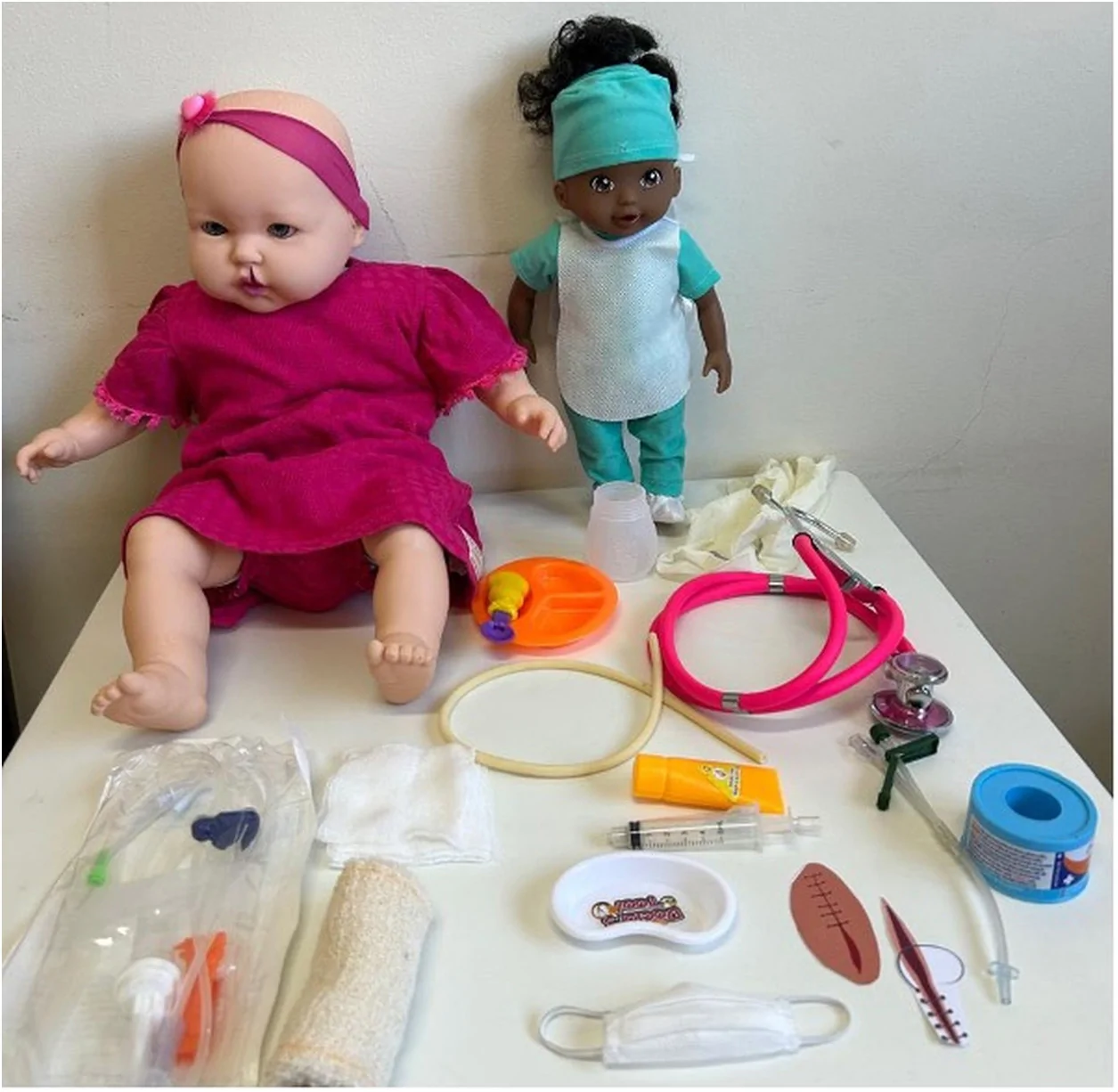
Toys used in the Instructional Therapeutic Play session.

**Source:** The authors.

The materials used belonged to the researcher/nurse and were selected according to their suitability for simulating the procedure. Although the institution provides multidisciplinary support services, ITP had not previously been incorporated into patient care, making this the first initiative of its kind.

During the ITP session, the child was encouraged to ask questions, all of which were answered by the interviewer. The discussion included topics related to anesthesia, venipuncture, the use of medical devices during surgery, and the operating room environment. At the end of the consultation, two additional books were read. The first, “*Você já tem uma marquinha?*”, a 20-page book, tells the story of a child with an orofacial cleft throughout the rehabilitation process^(10)^. The second, “*Quando me sinto feliz*”, a 15-page book, addresses the emotion of happiness^(11)^. With the consent of the child’s legal guardians, both the child’s voice and image were recorded throughout the consultation.

### Data Analysis and Processing

Data were analyzed descriptively using the consultation recording, nursing notes, and photographs taken during the session.

### Ethical Aspects

The study complied with all ethical requirements. It was approved by the Human Research Ethics Committee of the participating university under Opinion 7,041,250/2024 (Certificate of Presentation for Ethical Consideration 81990024.0.0000.0104). In addition, the Informed Consent Form and the Informed Assent Form were obtained in duplicate. The child’s legal guardians provided consent, and the child provided assent for the recording of her voice and image, as well as for the use of the drawing produced during the session, while ensuring anonymity and confidentiality.

## RESULTS

### Nursing Assessment

The patient was an 8-year-old girl diagnosed with a complete post-foramen cleft. She was receiving speech-language therapy and educational support at the association and, at her mother’s request, had resumed psychological follow-up. She underwent her first palatoplasty in 2018, at 2 years of age, which resulted in a fistula that had not yet been repaired.

The patient was an 8-year-old girl diagnosed with a complete post-foramen cleft. She was receiving speech-language therapy and educational support at the association and, at her mother’s request, had resumed psychological follow-up. She underwent her first palatoplasty in 2018, at 2 years of age, which resulted in a fistula that had not yet been repaired.

Initially, an individual nursing consultation was conducted with the child’s mother, who reported having experienced a traumatic situation during her daughter’s first surgical procedure because of fear and anxiety caused by delays in the surgery. Upon learning that her daughter would undergo another operation, the mother again became apprehensive. She was therefore provided with guidance regarding hospital rules and routines, the surgical procedure, and the importance of remaining calm, as caregivers represent the child’s primary source of security and reassurance, and children’s behavior often reflects that of their parents^(12)^.

An individual consultation was then conducted with the child using play-based strategies to facilitate verbal and nonverbal expression and communication. Initially, the book about fear^(9)^ was read. At that moment, the child expressed her feelings by stating: “*I liked it* (the story). *You have to face your fears, and I’m afraid of surgery*”.

To better understand the child’s fears regarding the procedure, she was then asked to produce a drawing in response to the question, “What are you afraid of about the surgery?”. After completing the drawing, she was invited to explain it, stating: “*I drew myself and the nurse. She’s letting out some smoke. I’m afraid of passing out and never waking up again. I don’t know why, but I’m afraid because then I won’t be alive anymore. I’m only afraid of going into the operating room. I’ve been there once, but I don’t remember it because I was a baby*”. The drawing is shown in Figure 2.

**Figure 2:**
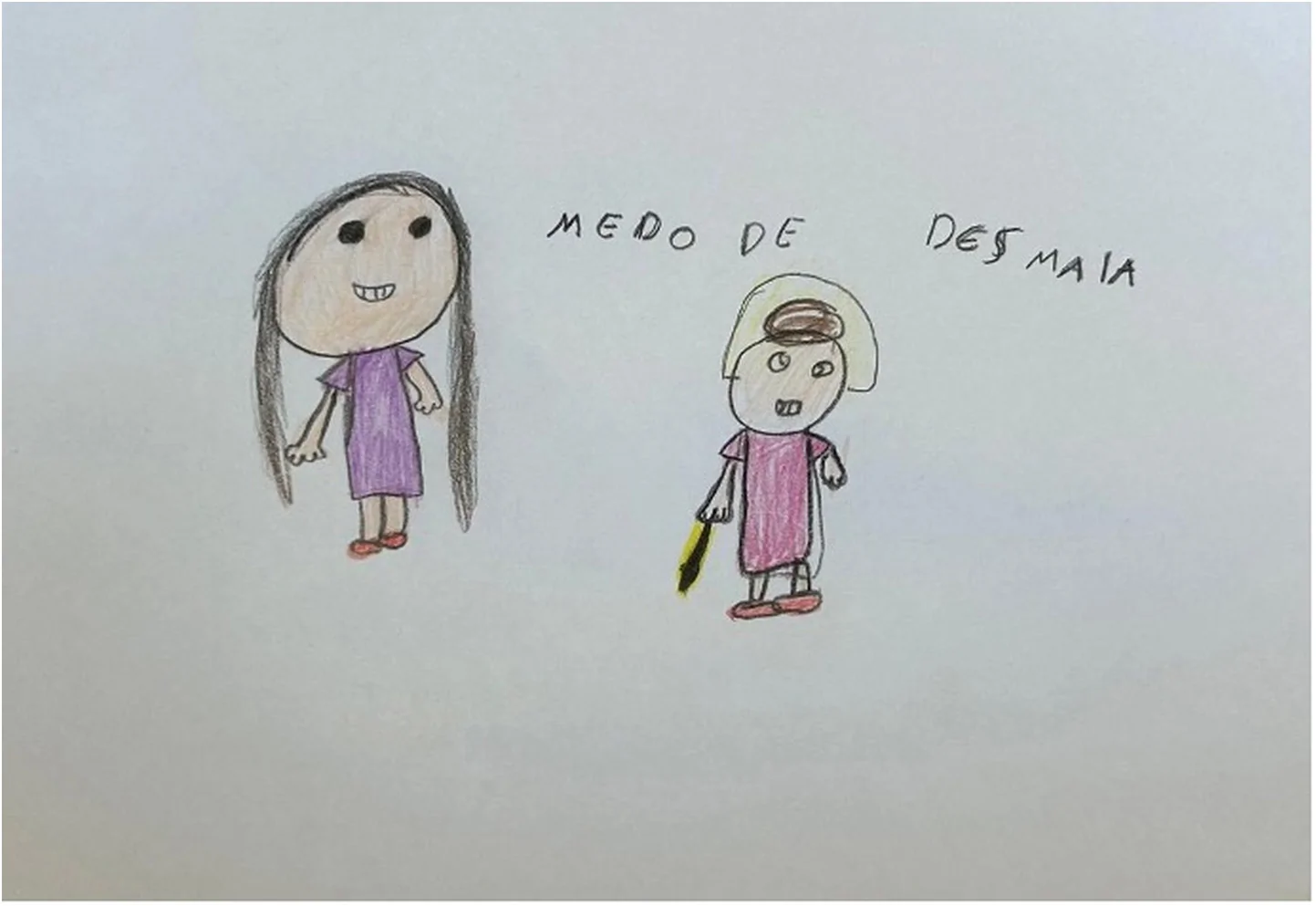
Drawing made by a child about the fear of surgery.

**Source:** Research data.

The child actively participated throughout the session, expressing all her questions and concerns. When the toys were used to demonstrate that peripheral venipuncture would be necessary, she asked: “*Will she poke me after I’m asleep? Will they leave it* (the needle) *there until the surgery is over?*” She also expressed anxiety related to anesthesia: “*Will they use the little smoke to make me sleep* (in the operating room)? *When they put the little smoke on me, will I fall asleep right away? Will it take long for me to fall asleep?*”.

Another question raised by the child concerned whether there would be other children at the hospital or whether she would be alone: “*Will there be other children there where I’ll be?*”. Furthermore, she associated the doll with a sense of security during the procedure and asked: “*Will she* (the doll) *be allowed to stay with me in the operating room?*”

At the end of the session, the child demonstrated that she understood the purpose of the surgery for her rehabilitation by stating: “*I won’t be able to see* (the stitches). *I’ll only notice it through my voice, right?*” (expected change and improvement in her voice after surgery). She also shared her opinion about the ITP session: “*I liked it* (the ITP session). *I’m not scared anymore. I think my mom will help keep me calm* (on the day of the surgery)”.

At the end of the session, the book telling the story of a child with an orofacial cleft and the child’s treatment journey was read^(10)^. Subsequently, another book about happiness was also read^(11)^. Afterward, the child expressed positive feelings about the session by stating: “(When I have the surgery), *I’ll be happy because I did it*”. She also recognized that the TP session had helped calm her: “*I’m less afraid now. I feel calmer*”.

### Nursing Diagnoses

Based on the ITP session and after identifying the child’s needs and problems, four nursing diagnoses were established according to NANDA International, as presented in Chart 1 ^(13)^.

**Chart 1:** Nursing diagnoses – Maringá, PR, Brazil, 2025.

Domain	Nursing diagnosis	Defining characteristics	Related factors
Domain 9 – Coping/Stress Tolerance	Excessive Anxiety	Excessive insecurity, nervousness, and tension	Unfamiliar situation (surgery) associated with invasive procedures
Domain 9 – Coping/Stress Tolerance	Excessive Fear	Apprehension, decreased self-confidence, and nervousness	Unfamiliar situation (surgery); children as an at-risk population; associated with invasive procedures
Domain 9 – Coping/Stress Tolerance	Readiness for Enhanced Coping	Desire to improve stress management	
Domain 9 – Coping/Stress Tolerance	Readiness for Enhanced Resilience	Desire to improve positive outlook, sense of control, and coping skills	

### Nursing Implementation

Based on the nursing diagnoses, the following nursing interventions were established according to the Nursing Interventions Classification^(14)^, as presented in Chart 2.

**Chart 2:** Nursing prescriptions – Maringá, PR, Brazil, 2025.

Nursing diagnosis	Nursing prescriptions
Excessive Anxiety	– Explain the surgical procedure and hospital routines to the child. – Provide information about the equipment that will be used. – Use toys to simulate procedures. – Use Instructional Therapeutic Play for preoperative preparation. – Continuously assess signs of anxiety before and after the interventions.
Excessive Fear	– Encourage the child to express feelings and perceptions related to fear. – Validate the child’s feelings and reassure her that feeling afraid is normal. – Provide a safe and welcoming environment. – Use Therapeutic Play to facilitate the expression of feelings related to the surgical procedure. – Monitor and reassess the intensity of fear throughout follow-up.
Readiness for Enhanced Coping	– Explore feelings and concerns related to the current situation. – Monitor the child’s progress in using coping strategies.
Readiness for Enhanced Resilience	– Promote hope and optimism through positive examples. – Assess the maintenance of positive attitudes toward the surgical procedure.

## DISCUSSION

The present case demonstrated manifestations of anxiety and fear associated with the surgical procedure, which are commonly observed among children with orofacial clefts. These children often require multiple surgical interventions throughout their growth and development, which may trigger negative emotional responses such as fear, anxiety, and panic, particularly when previous traumatic healthcare experiences have occurred^(15)^.

A study conducted in a Brazilian hospital found that incorporating TP into clinical practice produced positive outcomes for healthcare professionals, the institution, and the families of children admitted to a Pediatric Intensive Care Unit, demonstrating the transformative potential of this intervention. To implement TP in clinical practice, barriers to its use were identified, improvement strategies were planned, goals were established, a nurse leader was appointed, partnerships among healthcare professionals were developed, staff training was provided, and a reference group and institutional protocol for TP were created^(16)^.

A quasi-experimental study conducted in Brazil assessing children’s comfort found that the use of ITP promoted cooperative behavior, reduced nervousness by 43%, decreased movement and protest behavior by 51%, and increased helping behavior by 27% among children undergoing peripheral intravenous catheterization^(7)^.

The findings of the present study are consistent with the literature, demonstrating that ITP plays an important role in preparing children for surgery by increasing their confidence and reducing fear of the procedure. Likewise, another study conducted in Turkey used hand puppets with preschool children during blood collection and found that this strategy reduced both fear and pain during the procedure^(17)^.

Therefore, strategies that promote children’s comfort, confidence, and sense of security should be incorporated into care while taking into account the specific needs of each developmental stage. In this context, TP can be incorporated across all pediatric healthcare settings with the involvement of the entire multidisciplinary team^(18)^.

Accordingly, pediatric nursing professionals are supported by Federal Nursing Council Resolution 546/2017 to use play and TP in the care of hospitalized children and their families. According to this resolution, this technique should be incorporated into all stages of the nursing process and documented in the patient’s medical record^(19)^.

Furthermore, the nursing diagnoses identified reflected the child’s needs in relation to the surgical procedure. The diagnoses “Excessive Anxiety” and “Excessive Fear” reflected the child’s feelings of insecurity and fear regarding anesthesia^(13)^. Anxiety and fear are often assessed subjectively in pediatric surgical patients. A study conducted with Brazilian nurses found that providing accurate information during preoperative preparation is essential for reducing patients’ fear and anxiety throughout their stay in the operating room^(20)^.

The diagnoses “Readiness for Enhanced Coping” and “Readiness for Enhanced Resilience” demonstrated that children are capable of developing effective coping strategies when cared for in a playful and sensitive manner^(13)^. The use of play-based resources enables children to express their emotions and feel safer, thereby promoting greater self-control and confidence, which are essential for coping and resilience during childhood^(21)^.

The study has some limitations. It involved only a single session to prepare the child for surgery and did not include postoperative follow-up, which would have allowed a better understanding of the long-term benefits of the intervention. Moreover, because this is a single-case study conducted at only one support association, the generalizability of the findings is limited. However, this can be explained by the association’s routine schedule and by the child’s and caregivers’ failure to attend the follow-up appointments that had been scheduled.

Despite these limitations, the study offers valuable contributions to nursing practice by supporting the incorporation of TP into nursing consultations, thereby strengthening pediatric nursing care. In addition, the findings provide strategies and resources that may be implemented in other pediatric settings aimed at preparing children and reducing anxiety during healthcare experiences.

## CONCLUSION

This study demonstrated that incorporating ITP into the nursing consultation was an effective strategy for preparing children with orofacial clefts for surgery. The case report showed that the intervention enabled the child to express feelings of fear and anxiety while promoting a better understanding of the surgical procedure.

The findings reinforce the need to implement ITP as a systematic strategy in pediatric nursing practice, particularly in the preparation of children for invasive procedures, thereby contributing to more humanized care.

## Data Availability

The entire dataset supporting the results of this study was published in the article itself.

## References

[1] Vieira KM, Medeiros AK, Almeida JL, Silva Fo OB, Oliveira JFA (2025). Fendas orofaciais em recém-nascidos no Brasil: estudo de série temporal, 2010-2021. Epidemiol Serv Saude.

[2] Urményi GL, Fernandes EC, Urményi LG (2024). Prevalence of cleft lip and palate in Brazil and its notification in the information system. Rev Bras Cir Plást.

[3] Gomes MI, Diniz CS, Andrade TI (2024). Cuidados pré e pós-operatórios de queiloplastia e palatoplastia em crianças com fissura labiopalatina: revisão de literatura. Rev Multidiscip Nordeste Mineiro.

[4] Kim EN, Yang HJ, Shin JH, Chung YS, Lee YH, Baek SH (2023). Multidisciplinary presurgical education: clinical impact on children with orofacial clefts undergoing maxillary distraction via rigid external distraction. Cleft Palate Craniofac J.

[5] Arnaldo PRO, Gomes IM, Silva LSC, Martins JF, Oliveira LL (2023). Brinquedo terapêutico para crianças com cateter venoso central totalmente implantado: percepção dos enfermeiros. Texto Contexto Enferm.

[6] Miranda CB, Maia EBS, Almeida FA (2024). Perspectives of BrinquEinstein healthcare professionals on the implementation of therapeutic play in pediatrics. Cien Saude Colet.

[7] Santos LM, Rodrigues JF, Pereira ALA, Costa R (2025). Efeito do brinquedo terapêutico instrucional no comportamento de crianças na primeira tentativa de cateterização intravenosa. Rev Gaúcha Enferm.

[8] Yoshida WB (2007). Redação do relato de caso [editorial]. J Vasc Bras.

[9] Misse J (2021). Quando eu sinto medo.

[10] Zecchin KG (2022). Você já tem uma marquinha?.

[11] Misse J (2021). Quando me sinto feliz.

[12] Ma X, Zhang Z, Bao Y, Zhao H (2025). Impact of pediatric surgery on anxiety in children and their families and coping strategies: a narrative review. Transl Pediatr.

[13] Herdman TH, Kamitsuru S, Lopes CT (2024). Diagnósticos de enfermagem da NANDA-I: definições e classificação – 2024-2026.

[14] Butcher HK, Bulechek GM, Dochterman JM, Wagner CM (2020). Classificação das intervenções de enfermagem (NIC).

[15] Van Roey VL, Irvine WFE (2024). Optimal diagnostic and treatment practices for facial dysostosis syndromes: a clinical consensus statement among European experts. J Craniofac Surg.

[16] Miranda CB, Maia EBS, Almeida FA (2022). Modelo de implementação sistemática do brinquedo terapêutico em unidades pediátricas hospitalares. Esc Anna Nery.

[17] Oluç T, Sarialiogˇlu A (2023). The effect of a hand puppet-based therapeutic play for preschool children on the fear and pain associated with blood collection procedure. J Pediatr Nurs.

[18] Mandetta MA, Toso BRGO, Gaiva MAM, Maia EBS, Barber ROLB, Ribeiro CA (2023). Brincar e o brinquedo terapêutico: na assistência de enfermagem à criança e família.

[19] Brasil. Conselho Federal de Enfermagem (2017). Resolução COFEN nº 546, de 9 de maio de 2017. Atualiza norma para utilização da técnica do brinquedo/brinquedo terapêutico pela equipe de enfermagem na assistência à criança hospitalizada [Internet].

[20] Jerez-Molina C, Lahuerta-Valls L, Fernandez-Villegas V, Santos-Ruiz S (2023). Nursing evaluation of pediatric preoperative anxiety: a qualitative study. Rev Lat Am Enfermagem.

[21] Díaz-Rodríguez M, Alcántara-Rubio L, Aguilar-García D, Pérez-Muñoz C, Carretero-Bravo J, Puertas-Cristóbal E (2021). The effect of play on pain and anxiety in children in the field of nursing: a systematic review. J Pediatr Nurs.

