# Supplementary material for: Use of Instructional Therapeutic Play during the preoperative nursing consultation of a child with orofacial clefts
Source: Rev Esc Enferm USP. 2026 Oct 12;60:e20260112. doi: 10.1590/1980-220X-REEUSP-2026-0112en (PMC13682276; doi:10.1590/1980-220X-REEUSP-2026-0112en)
Supplement: Translation

# Translation: Uso do Brinquedo Terapêutico Instrucional na consulta de enfermagem pré-operatória de criança com fissura orofacial

**DOI:** 10.1590/1980-220X-REEUSP-2026-0112pt

**Authors:** Mariana Martire Mori, Camila Moraes Garollo Piran, Alana Vitória Escritori Cargnin, Laís Moreira Martins, Maricy Morbin Torres, Marcela Demitto Furtado

**Affiliations:** 1 Universidade Estadual de Maringá, Programa de Pós-Graduação em Enfermagem, Maringá, PR, Brasil.; 2 Universidade Estadual de Maringá, Departamento de Enfermagem, Maringá, PR, Brasil.

**Keywords:** Brincadeiras e Brinquedos, Cuidados Pré-Operatórios, Enfermagem Pediátrica, Fissura Palatina, Processo de Enfermagem

## Abstract

**Objetivo:**

Relatar o uso do Brinquedo Terapêutico Instrucional na consulta de enfermagem pré-operatória de uma criança com fissura orofacial

**Método:**

Estudo de caso desenvolvido em uma associação de apoio a crianças com fissura orofacial, localizada no noroeste do Paraná, Brasil. Os dados foram coletados em maio de 2025, durante uma consulta de enfermagem, com o uso do Brinquedo Terapêutico Instrucional, da leitura de livros e do desenho, com vistas ao preparo cirúrgico da criança. O atendimento foi gravado sob consentimento, e os dados foram analisados de forma descritiva. A pesquisa foi aprovada pelo Comitê de Ética em Pesquisa, sob Parecer n^o^ 7.041.250/2024.

**Resultados:**

Incluiu-se uma criança de 8 anos, do sexo feminino, com cirurgia programada para a correção de palato. Apresentava medo e ansiedade relacionados à cirurgia, principalmente o medo de não acordar da anestesia. O uso do Brinquedo Terapêutico Instrucional permitiu que a criança compreendesse o procedimento, expressasse as suas dúvidas e, ao final da sessão, relatou estar mais tranquila.

**Conclusão:**

O Brinquedo Terapêutico Instrucional favoreceu a expressão emocional e o preparo cirúrgico da criança com fissura orofacial.

## INTRODUÇÃO

As fendas orofaciais são malformações congênitas que afetam a região da face e resultam de falhas no desenvolvimento do lábio e/ou do palato primário e secundário durante as fases iniciais da formação embrionária. Essas anomalias podem comprometer, em diferentes graus, o lábio, a cavidade oral e a cavidade nasal. De acordo com sua extensão, elas são classificadas em três grandes grupos: fenda palatina, fenda labial, e fenda labiopalatina. Podem se manifestar de forma isolada, o que corresponde a cerca de 70% dos casos, ou estar associadas a uma ampla variedade de síndromes genéticas, representando os 30% restantes^(1)^.

No Brasil, a incidência estimada de fendas orofaciais é de aproximadamente um caso para cada 650 nascimentos, o que corresponde a uma taxa de 1,53 por 1.000 nascidos vivos. Outras tentativas de mensuração dessa prevalência têm sido limitadas por fatores metodológicos, como o uso de amostras reduzidas e a dependência de registros hospitalares locais, o que compromete a confiabilidade e a generalização dos dados^(2)^.

O procedimento cirúrgico para a correção da malformação deve ser realizado ainda no primeiro ano de vida, evitando consequências prejudiciais a determinadas funções do paciente, como a fala e a deglutição, com o objetivo de garantir uma melhor qualidade de vida para as crianças acometidas por essas anomalias. A atuação integrada de uma equipe multiprofissional é fundamental para a eficácia do manejo terapêutico das fissuras orofaciais, considerando a complexidade anatômica, funcional e psicossocial envolvida nesse tipo de condição^(3)^.

Nesse sentido, sessões educativas pré-operatórias conduzidas por equipes multidisciplinares resultam em benefícios significativos para crianças submetidas a procedimentos cirúrgicos, incluindo a redução no tempo de internação e no uso de analgésicos^(4)^. Diante do exposto, é inerente à prática do enfermeiro pediátrico a busca por estratégias que promovam o engajamento ativo da criança no processo de cuidado e que contribuam para a redução de possíveis traumas decorrentes das intervenções em saúde^(5)^.

Uma alternativa para a promoção da participação efetiva da criança é a utilização do Brinquedo Terapêutico (BT), cujos benefícios são amplamente documentados na literatura científica, o que enfatiza sua relevância para o desenvolvimento e o bem-estar da criança, recomendando sua implementação sistemática nos diferentes contextos de atenção à saúde infantil^(6)^.

Entre as modalidades existentes, o Brinquedo Terapêutico Instrucional (BTI) é a escolha mais pertinente para a preparação da criança para a hospitalização e procedimentos terapêuticos^(5)^. As evidências confirmam que a aplicação do BT no cuidado da criança hospitalizada traz contribuições positivas na redução da ansiedade e do medo das crianças e de seus familiares, cooperando para o desenvolvimento de uma assistência de enfermagem mais humanizada^(6)^.

Existem poucos estudos que evidenciam o efeito do BTI em procedimentos invasivos em crianças. No entanto, os enfermeiros pediatras desempenham um papel central na condução e na implementação de estudos clínicos inovadores voltados à investigação das repercussões do brincar terapêutico na promoção da saúde infantil e na atenuação de comportamentos associados ao estresse, os quais podem intensificar vivências potencialmente traumáticas durante o cuidado em saúde^(7)^. Diante do exposto, o objetivo deste estudo clínico é relatar o uso do BTI na consulta de enfermagem pré-operatória de uma criança com fissura orofacial.

## MÉTODO

### Tipo de Estudo

Trata-se de um estudo do tipo relato de caso único a respeito da aplicação do BTI durante uma consulta de enfermagem para o preparo cirúrgico de uma criança com fissura orofacial. O relato de caso é uma fonte importante de informação, que contribui para a melhoria do atendimento e do tratamento, principalmente quando aponta estratégias de cuidado ainda pouco utilizadas na prática^(8)^.

### Cenário

O estudo foi desenvolvido em uma associação de apoio localizada em um município no noroeste do estado do Paraná, Brasil, que realiza atendimento multiprofissional gratuito a crianças e adolescentes com fissura orofacial. Atualmente, são atendidos aproximadamente 500 pacientes que residem em 80 municípios da região. Atuam na instituição profissionais das áreas da psicologia, nutrição, fonoaudiologia, odontologia, serviço social e apoio pedagógico. As demais especialidades que integram a atenção às crianças com fissura orofacial prestam atendimento em um hospital localizado em outro município no leste do Paraná, onde as crianças são encaminhadas para realizar os procedimentos cirúrgicos.

### Critérios de Seleção

O relato do caso foi produzido com base em uma consulta de enfermagem na qual foram utilizados o BTI e estratégias lúdicas com uma criança com fissura orofacial. A paciente foi atendida por uma enfermeira que é mestranda em enfermagem e realiza um projeto de pesquisa na instituição, após o encaminhamento da psicóloga do serviço, que observou o medo da criança de realizar o procedimento cirúrgico.

### Coleta de Dados

Os dados foram coletados em um único atendimento, realizado em maio de 2025, no mês que antecedeu o procedimento cirúrgico, de forma individual e na própria associação. A consulta de enfermagem foi conduzida de maneira estruturada em dois momentos: o atendimento ao cuidador familiar e o atendimento à criança. A coleta de dados teve duração aproximada de 60 minutos, sendo 20 minutos destinados ao atendimento do cuidador familiar e 40 minutos ao atendimento da criança.

A princípio, a mãe da criança foi atendida, individualmente, para obter mais informações relacionadas ao contexto familiar e de saúde. Na sequência, foi realizado o atendimento da criança, iniciado com a leitura de um livro na íntegra intitulado “Quando eu sinto medo”, composto por 16 páginas, que aborda o reconhecimento e o enfrentamento do medo na infância^(9)^. Em seguida, foi solicitado que a criança fizesse um desenho a partir da pergunta “Qual é o seu medo de realizar a cirurgia?”. Ao término, a criança explicou o que significava o seu desenho.

Dando continuidade às etapas da consulta de enfermagem, a enfermeira realizou a sessão de BTI como estratégia de avaliação e intervenção de enfermagem no contexto da consulta para demonstrar como seria o procedimento cirúrgico. Foram utilizados diversos brinquedos que mais se aproximavam da realidade, incluindo uma boneca com fissura, uma boneca enfermeira, gaze, máscara, luva, cateter intravenoso periférico, estetoscópio, seringa, equipo extensor multivias, fita Micropore, garrote, equipo de medicação, adesivos de incisão cirúrgica, colher, prato e copo de brinquedo, conforme apresentado na Figura 1.

**Figura 1:** Brinquedos utilizados na sessão de Brinquedo Terapêutico Instrucional.

**Fonte:** os autores.

Os materiais utilizados eram da pesquisadora/enfermeira, selecionados conforme a adequação à simulação do procedimento. Ressalta-se que, embora a instituição desenvolva ações de apoio multiprofissional, o BTI não é utilizado nos atendimentos, sendo esse o trabalho inicial.

Durante a sessão de BTI, a criança pôde fazer perguntas à entrevistadora, que respondeu a todas, abordando temas relacionados ao procedimento anestésico, à punção venosa, à permanência de dispositivos durante a cirurgia e ao ambiente do centro cirúrgico. Ao término da consulta, foram lidos outros dois livros, sendo o primeiro intitulado “Você já tem uma marquinha?”, contendo 20 páginas, que contava a trajetória de uma criança com fissura orofacial durante sua reabilitação^(10)^. O segundo livro era intitulado “Quando me sinto feliz”, contendo 15 páginas, sobre o sentimento de felicidade^(11)^. Com o consentimento dos responsáveis, a voz e a imagem da criança foram gravadas durante todo o atendimento.

### Análise e Tratamento dos Dados

Os dados foram analisados de forma descritiva, utilizando a gravação do atendimento, as anotações de enfermagem e as fotos que foram tiradas.

### Aspectos Éticos

A pesquisa respeitou todas as diretrizes éticas. Foi aprovada pelo Comitê de Ética em Pesquisa com Seres Humanos da universidade signatária, sob Parecer n^o^ 7.041.250/2024 (Certificado de Apresentação para Apreciação Ética n^o^ 81990024.0.0000.0104). Além disso, foram aplicados o Termo de Consentimento Livre e Esclarecido e o Termo de Assentimento Livre e Esclarecido, ambos em duas vias. Houve consentimento dos responsáveis legais e assentimento da criança para a gravação de sua voz e imagem, bem como para a utilização do desenho produzido durante a sessão, preservando-se o anonimato e a confidencialidade das informações.

## RESULTADOS

### Avaliação de Enfermagem

A criança, de 8 anos de idade e do sexo feminino, foi diagnosticada com fissura pós-forame completa. Ela está em acompanhamento fonoaudiológico e apoio pedagógico em uma associação de apoio e, por solicitação da mãe, retornou ao acompanhamento psicológico. Ela realizou sua primeira cirurgia de palatoplastia em 2018, aos dois anos de idade, que ocasionou uma fístula ainda não corrigida.

A criança estava com cirurgia de correção de fístula prevista para 2022, momento em que apresentou sintomas de ansiedade e o procedimento não foi realizado, mesmo passando por atendimento psicológico. A cirurgia foi agendada novamente para junho de 2025, e a criança voltou a apresentar sintomas de ansiedade e pânico do jaleco branco.

Inicialmente, foi realizado um atendimento de enfermagem individual com a mãe da criança, que relatou ter passado por uma experiência traumática no primeiro procedimento cirúrgico da filha, devido ao medo e à ansiedade causados pela demora no procedimento. Ao saber que a filha passaria por outra cirurgia, a mãe ficou apreensiva novamente. Dessa forma, foram dadas orientações à mãe sobre normas e rotinas hospitalares, sobre o procedimento e sobre a importância de manter a calma, pois os cuidadores são o ponto de referência e segurança para as crianças, e muitas vezes o comportamento delas é um reflexo do comportamento dos pais^(12)^.

Foi realizado um atendimento individual com a criança, utilizando estratégias lúdicas para facilitar sua expressão e comunicação verbal e não verbal. Inicialmente, foi feita a leitura de um livro sobre o medo^(9)^. Nesse momento, a criança já expressou seu sentimento a respeito, relatando: “*Eu achei legal* (a história). *Tem que enfrentar o medo, e eu tô com medo de cirurgia*”.

Em seguida, para compreender de fato qual o medo da criança em relação ao procedimento, foi solicitado que ela fizesse um desenho a partir da questão “Qual é o seu medo de realizar a cirurgia?”. Ao finalizar, foi solicitado que a criança explicasse o seu desenho, e ela relatou: “*Eu desenhei eu e a enfermeira. Ela tá soltando fumacinha. Eu tenho medo de desmaiar e nunca mais acordar, eu não sei porque, mas tenho medo, porque aí eu não vou viver. Eu só tenho medo de entrar no centro cirúrgico. Eu já fui uma vez, mas eu não lembro, porque eu era bebê*”. O desenho está representado na Figura 2.

**Figura 2:** Desenho produzido pela criança sobre o medo da cirurgia.

**Fonte:** dados da pesquisa.

A criança interagiu de forma ativa durante toda a sessão, expressando todas as suas dúvidas e anseios. Ao demonstrar, com os brinquedos, que seria necessária uma punção venosa a criança expressou: “*Ela vai me furar quando eu tiver dormindo? Eles vão deixar lá* (a agulha) *até a cirurgia acabar?*”. A criança também revelou a ansiedade relacionada à anestesia: “*Eles vão fazer a fumacinha pra eu dormir* (no centro cirúrgico). *Quando ela colocar a fumacinha, eu já vou dormir na hora? Vai demorar pra eu dormir?*”.

Outra curiosidade expressada foi saber se teriam mais crianças no hospital ou se ela estaria sozinha: “*Lá onde eu vou estar, vai ter mais crianças também?*”. Além disso, a boneca foi associada à segurança para a criança durante o procedimento, e ela questionou: “*Ela* (boneca) *vai poder ficar no centro cirúrgico comigo?*”.

Ao final da sessão, a criança compreendeu o objetivo do procedimento cirúrgico para a sua reabilitação: “*Eu não vou conseguir ver* (o ponto). *Eu só vou conseguir ver pela voz mesmo, né?* (mudança e melhora na voz após a cirurgia)”. Ainda, expressou a sua opinião sobre a sessão de BT *“Eu* achei (sessão de BTI) *legal, não tô mais com medo. Acho que minha mãe vai me deixar calma* (no dia da cirurgia)”.

Ao final da sessão, foi realizada a leitura de um livro que conta a história de uma criança com fissura orofacial e a sua rotina de tratamento^(10)^. Posteriormente, também foi realizada a leitura de um livro sobre felicidade^(11)^. Ao término, a criança expressou sentimentos positivos em relação à sessão: “(quando fizer a cirurgia) *eu vou me sentir feliz por ter conseguido*”. Ainda, percebeu que a sessão de BT foi importante para acalmá-la: “*Eu tô com menos medo, eu me tranquilizei*”.

### Diagnósticos de Enfermagem

A partir do BTI, ao identificar as necessidades e os problemas da criança, foram elencados quatro diagnósticos de enfermagem, de acordo com a NANDA-*International*, conforme o Quadro 1 ^(13)^.

**Quadro 1:** Diagnósticos de enfermagem – Maringá, PR, Brasil, 2025.

Domínio	Diagnóstico de enfermagem	Características definidoras	Fatores relacionados
Domínio 9 – Enfrentamento/tolerância ao estresse	Ansiedade Excessiva	Insegurança, nervosismo e tensão	Situação desconhecida (cirurgia), associada a procedimentos invasivos
Domínio 9 – Enfrentamento/tolerância ao estresse	Medo Excessivo	Apreensão, auto segurança diminuída e nervosismo	Situação desconhecida (cirurgia) e a população de risco de crianças, associado a procedimentos invasivos
Domínio 9 – Enfrentamento/tolerância ao estresse	Disposição para Enfrentamento Melhorado	Desejo de melhorar a o manejo do estresse	
Domínio 9 – Enfrentamento/tolerância ao estresse	Disposição para Resiliência Melhorada	Desejo de melhorar a perspectiva positiva, o senso de controle e habilidades de enfrentamento	

### Implementação da Enfermagem

Dessa forma, foram estabelecidas as seguintes possíveis intervenções de enfermagem, de acordo com a Classificação das Intervenções de Enfermagem^(14)^, presentes no Quadro 2.

**Quadro 2:** Prescrições de enfermagem – Maringá, PR, Brasil, 2025.

Diagnóstico de enfermagem	Prescrições de enfermagem
Ansiedade Excessiva	– Explicar o procedimento e as rotinas hospitalares à criança. – Informar sobre os equipamentos que serão utilizados. – Utilizar brinquedos para simular procedimentos. – Utilizar o Brinquedo Terapêutico Instrucional para preparo pré-operatório. – Avaliar continuamente os sinais de ansiedade antes e após as intervenções.
Medo Excessivo	– Incentivar a expressão de sentimentos e percepções sobre o medo. – Validar os sentimentos da criança e assegurar que é normal sentir medo. – Proporcionar um ambiente seguro e acolhedor. – Aplicar o Brinquedo Terapêutico para expressão de sentimentos relacionados ao procedimento cirúrgico. – Monitorar e reavaliar a intensidade do medo ao longo do acompanhamento.
Disposição para Enfrentamento Melhorado	– Explorar sentimentos e preocupações relacionadas à situação atual. – Acompanhar a evolução das estratégias de enfrentamento adotadas pela criança.
Disposição para Resiliência Melhorada	– Promover a esperança e o otimismo através de exemplos positivos. – Avaliar a manutenção de atitudes positivas frente ao procedimento cirúrgico.

## DISCUSSÃO

O caso em questão apresenta manifestações de ansiedade e medo associadas ao procedimento cirúrgico, aspectos frequentemente relacionados a vivências comuns na vida de crianças com fissuras orofaciais. Essas crianças necessitam de diversas intervenções cirúrgicas ao longo do crescimento e desenvolvimento, o que pode despertar sensações emocionais negativas, como medo, ansiedade e pânico, especialmente se já possuírem um contexto e experiências prévias traumáticas^(15)^.

Um estudo conduzido em um hospital do Brasil identificou que a inserção do BT na assistência trouxe resultados positivos para a prática dos profissionais, para a instituição e para os familiares de crianças internadas em uma Unidade de Terapia Intensiva, apontando que o uso do brinquedo é transformador. Para implementar o brinquedo na assistência, foram identificadas as barreiras para o seu uso, planejadas melhorias, definidas metas, identificado um enfermeiro líder, estabelecidas parcerias entre profissionais, promovidos treinamentos para a equipe e criado um grupo de referência para o BT e um protocolo institucional^(16)^.

Um estudo quase-experimental realizado no Brasil, ao considerar o conforto das crianças, verificou que o uso do BTI favoreceu o comportamento colaborativo delas e foi responsável por reduzir em 43% o nervosismo, em 51% a movimentação e o não protesto, e por aumentar em 27% o comportamento auxiliar em crianças submetidas à cateterização intravenosa periférica^(7)^.

Os achados deste estudo são consistentes com a literatura, pois evidenciam a importância do BTI no preparo da criança, aumentam a confiança e reduzem o medo do procedimento. Ademais, outra pesquisa conduzida na Turquia utilizou fantoches de mão com crianças pré-escolares durante o procedimento de coleta de sangue, e os resultados determinaram que a estratégia reduziu o medo e a dor das crianças durante o procedimento^(17)^.

Nesse sentido, considera-se a necessidade de incorporar estratégias que promovam o conforto, a confiança e a segurança da criança, levando em conta as necessidades especiais de cada faixa etária. Dessa forma, o BT pode ser incorporado em todos os contextos de atenção à saúde da criança, com a integração de toda a equipe multiprofissional^(18)^.

Destarte, a equipe de enfermagem que atua na pediatria é respaldada através da Resolução nº 546/2017 do Conselho Federal de Enfermagem para a utilização do brinquedo e do BT na assistência à criança e aos familiares hospitalizados. De acordo com a resolução, essa técnica deve contemplar as etapas do processo de enfermagem e deve constar em registro no prontuário^(19)^.

Além disso, foi possível identificar diagnósticos de enfermagem que refletiram as necessidades da criança diante do procedimento cirúrgico. Os diagnósticos “Ansiedade Excessiva” e “Medo Excessivo” revelam os sentimentos de insegurança e medo da anestesia^(13)^. A ansiedade e o medo, muitas vezes, são avaliados de forma subjetiva em pacientes pediátricos cirúrgicos. Um estudo conduzido com enfermeiros brasileiros identificou que, para esses profissionais, fornecer informações fidedignas ao paciente durante o preparo pré-operatório é crucial para reduzir o medo e a ansiedade durante o tempo de permanência no centro cirúrgico^(20)^.

Já os diagnósticos “Disposição para Enfrentamento Melhorado” e “Disposição para Resiliência Melhorada” evidenciam que a criança é capaz de desenvolver estratégias de enfrentamento quando acolhida de forma lúdica e sensível^(13)^. O uso de recursos lúdicos possibilita à criança expressar emoções e sentir-se mais segura, contribuindo para o aumento do autocontrole e da confiança, aspectos essenciais para o enfrentamento e a resiliência infantil^(21)^.

O estudo apresenta limitações que estão relacionadas à realização de apenas uma sessão para o preparo da criança ao procedimento cirúrgico e à ausência de seguimento no pós-operatório, o que permitiria compreender melhor os benefícios da intervenção a longo prazo. Além disso, por se tratar de um estudo de caso, realizado em uma única associação de apoio, há limitação quanto à generalização dos achados. No entanto, esse fato se justifica devido à rotina de atendimentos da associação e ao não comparecimento da criança e dos seus responsáveis aos atendimentos que foram agendados.

Apesar disso, o estudo oferece aplicações valiosas para a prática de enfermagem, corroborando a incorporação do BT nas consultas de enfermagem, o que fortalece a assistência pediátrica. Além disso, a pesquisa oferece subsídios e recursos que podem ser implementados em outros contextos pediátricos que objetivam preparar a criança e reduzir a ansiedade no contexto de saúde.

## CONCLUSÃO

O estudo evidenciou que incorporar o BTI na consulta de enfermagem foi uma estratégia eficaz na preparação cirúrgica de crianças com fissuras orofaciais. O relato do caso demonstrou que a intervenção permitiu que a criança expressasse os sentimentos de medo e ansiedade, favorecendo o esclarecimento sobre o procedimento.

O estudo reforça a necessidade da implementação do BTI como estratégia sistematizada na prática de enfermagem pediátrica, especialmente no preparo para procedimentos invasivos, contribuindo para a humanização do cuidado.

## Data Availability

Todo o conjunto de dados que dá suporte aos resultados deste estudo foi publicado no próprio artigo.

# Translation: Uso de Juegos Terapéuticos Instruccionales en la consulta de enfermería preoperatoria de una niña con fisura orofacial

**DOI:** 10.1590/1980-220X-REEUSP-2026-0112es

**Authors:** Mariana Martire Mori, Camila Moraes Garollo Piran, Alana Vitória Escritori Cargnin, Laís Moreira Martins, Maricy Morbin Torres, Marcela Demitto Furtado

**Affiliations:** 1 Universidade Estadual de Maringá, Programa de Pós-Graduação em Enfermagem, Maringá, PR, Brasil.; 2 Universidade Estadual de Maringá, Departamento de Enfermagem, Maringá, PR, Brasil.

**Keywords:** Juego e Implementos de Juego, Cuidados Preoperatorios, Enfermería Pediátrica, Fisura del Paladar, Proceso de Enfermería

## Abstract

**Objetivo:**

Informar sobre el uso del Juego Terapéutico Instruccional en la consulta de enfermería preoperatoria de una niña con fisura orofacial.

**Método:**

Estudio de caso realizado en una asociación de apoyo a niños con fisura orofacial, ubicada en el noroeste de Paraná, Brasil. Los datos se recopilaron en mayo de 2025, durante una consulta de enfermería, utilizando Juego Terapéutico Instruccional, lectura de libros y dibujo, con el fin de preparar al niño para la cirugía. La consulta se grabó con el consentimiento informado y los datos se analizaron de forma descriptiva. La investigación fue aprobada por el Comité de Ética en Investigación, bajo el Dictamen n^o^ 7.041.250/2024.

**Resultados:**

Se incluyó a una niña de 8 años programada para cirugía de corrección de paladar. Presentaba miedo y ansiedad relacionados con la cirugía, principalmente el miedo a no despertar de la anestesia. El uso del Juego Terapéutico Instruccional le permitió comprender el procedimiento, expresar sus dudas y, al finalizar la sesión, manifestó sentirse más tranquila.

**Conclusión:**

El Juego Terapéutico Instruccional facilitó la expresión emocional y la preparación quirúrgica en la niña con fisura orofacial.

## INTRODUCCIÓN

Las fisuras orofaciales son malformaciones congénitas que afectan la región facial y son el resultado de fallas en el desarrollo del labio y/o del paladar primario y secundario durante las fases iniciales de la formación embrionaria. Estas anomalías pueden afectar, en distintos grados, al labio, la cavidad oral y la cavidad nasal. Según su extensión, se clasifican en tres grandes grupos: fisura palatina, fisura labial y fisura labiopalatina. Pueden manifestarse de forma aislada, lo que corresponde a alrededor del 70 % de los casos, o estar asociadas a una amplia variedad de síndromes genéticos, lo que representa el 30 % restante^(1)^.

En Brasil, la incidencia estimada de fisuras orofaciales es de aproximadamente un caso por cada 650 nacimientos, lo que corresponde a una tasa de 1,53 por cada 1 000 nacidos vivos.

Otros intentos de medir esta prevalencia se han visto limitados por factores metodológicos, como el uso de muestras reducidas y la dependencia de registros hospitalarios locales, lo que compromete la confiabilidad y la generalización de los datos^(2)^.

El procedimiento quirúrgico para corregir la malformación debe realizarse aún durante el primer año de vida, a fin de evitar consecuencias perjudiciales para ciertas funciones del paciente, como el habla y la deglución, con el objetivo de garantizar una mejor calidad de vida para los niños afectados por estas anomalías. La intervención integrada de un equipo multiprofesional es fundamental para la eficacia del manejo terapéutico de las fisuras orofaciales, dada la complejidad anatómica, funcional y psicosocial que implica este tipo de afección^(3)^.

En este sentido, las sesiones educativas preoperatorias dirigidas por equipos multidisciplinarios aportan beneficios significativos a los niños sometidos a procedimientos quirúrgicos, incluyendo la reducción del tiempo de hospitalización y del uso de analgésicos^(4)^. Ante lo expuesto, es inherente a la práctica del enfermero pediátrico la búsqueda de estrategias que promuevan la participación activa del niño en el proceso de atención y que contribuyan a reducir los posibles traumas derivados de las intervenciones en salud^(5)^.

Una alternativa para promover la participación efectiva del niño es el uso del Juguete Terapéutico (JT), cuyos beneficios están ampliamente documentados en la literatura científica, lo que resalta su relevancia para el desarrollo y el bienestar del niño, y recomienda su implementación sistemática en los diferentes contextos de atención de la salud infantil^(6)^.

Entre las modalidades existentes, el Juguete Terapéutico Instruccional (JTI) es la opción más adecuada para preparar al niño para la hospitalización y los procedimientos terapéuticos^(5)^. La evidencia confirma que la aplicación del Juego Terapéutico en la atención del niño hospitalizado aporta beneficios en la reducción de la ansiedad y el miedo de los niños y sus familiares, lo que contribuye al desarrollo de una atención de enfermería más humanizada^(6)^.

Existen pocos estudios que demuestren el efecto del Juego Terapéutico Instruccional en procedimientos invasivos en niños. Sin embargo, los enfermeros pediátricos desempeñan un papel central en la dirección y la implementación de estudios clínicos innovadores orientados a investigar las repercusiones del juego terapéutico en la promoción de la salud infantil y en la atenuación de comportamientos asociados al estrés, los cuales pueden intensificar experiencias potencialmente traumáticas durante la atención en salud^(7)^. Ante lo expuesto, el objetivo de este estudio clínico es informar sobre el uso del JTI en la consulta de enfermería preoperatoria de un niño con fisura orofacial.

## MÉTODO

### Tipo de Estudio

Se trata de un estudio de tipo informe de caso único sobre la aplicación del JTI durante una consulta de enfermería para la preparación quirúrgica de un niño con fisura orofacial. El informe de caso es una fuente importante de información que contribuye a la mejora de la atención y el tratamiento, especialmente cuando señala estrategias de cuidado aún poco utilizadas en la práctica^(8)^.

### Contexto

El estudio se llevó a cabo en una asociación de apoyo ubicada en un municipio al noroeste del estado de Paraná, Brasil, que brinda atención multiprofesional gratuita a niños y adolescentes con fisura orofacial. Actualmente, se atiende a aproximadamente 500 pacientes que residen en 80 municipios de la región. En la institución trabajan profesionales de las áreas de psicología, nutrición, fonoaudiología, odontología, servicio social y apoyo pedagógico. Las demás especialidades que forman parte de la atención a los niños con fisura orofacial brindan servicio en un hospital ubicado en otro municipio al este de Paraná, adonde se deriva a los niños para realizar los procedimientos quirúrgicos.

### Criterios de Selección

El informe de caso se elaboró a partir de una consulta de enfermería en la que se utilizaron el JTI y estrategias lúdicas con una niña con fisura orofacial. La paciente fue atendida por una enfermera que cursa una maestría en enfermería y realiza un proyecto de investigación en la institución, tras la derivación de la psicóloga del servicio, quien observó el miedo de la niña a someterse al procedimiento quirúrgico.

### Recolección de Datos

Los datos se recopilaron en una sola sesión de atención, realizada en mayo de 2025, el mes anterior al procedimiento quirúrgico, de manera individual y en la propia asociación. La consulta de enfermería se llevó a cabo de manera estructurada en dos momentos: la atención al cuidador familiar y la atención a la niña. La recolección de datos duró aproximadamente 60 minutos, de los cuales 20 minutos se dedicaron a la atención del cuidador familiar y 40 minutos a la atención del niño.

En primer lugar, se atendió a la madre del niño, de manera individual, para obtener más información relacionada con el contexto familiar y de salud. A continuación, se llevó a cabo la atención al niño, que comenzó con la lectura completa de un libro titulado “Cuando tengo miedo”, compuesto por 16 páginas, que aborda el reconocimiento y el manejo del miedo en la infancia^(9)^. Luego, se le pidió al niño que hiciera un dibujo a partir de la pregunta “¿Qué te da miedo de la cirugía?”. Al finalizar, la niña explicó qué significaba su dibujo.

Continuando con las etapas de la consulta de enfermería, la enfermera llevó a cabo la sesión de JTI como estrategia de evaluación e intervención de enfermería en el contexto de la consulta para demostrar cómo sería el procedimiento quirúrgico. Se utilizaron diversos juguetes que se acercaban lo más posible a la realidad, incluyendo una muñeca con fisura, una muñeca enfermera, gasas, una mascarilla, un guante, un catéter intravenoso periférico, un estetoscopio, una jeringa, un equipo extensor multivía, cinta Micropore, un torniquete, un equipo de medicación, adhesivos para incisiones quirúrgicas, una cuchara, un plato y un vaso de juguete, tal como se muestra en la Figura 1.

**Figura 1:** Juguetes utilizados en la sesión de Juego Terapéutico Instruccional.

**Fuente:** Los autores.

Los materiales utilizados pertenecían a la investigadora/enfermera y fueron seleccionados según su idoneidad para la simulación del procedimiento. Cabe destacar que, aunque la institución lleva a cabo acciones de apoyo multiprofesional, el JTI no se utiliza en las consultas, por lo que este es el trabajo inicial.

Durante la sesión de JTI, la niña pudo hacerle preguntas a la entrevistadora, quien respondió a todas ellas, abordando temas relacionados con el procedimiento anestésico, la punción venosa, la permanencia de dispositivos durante la cirugía y el ambiente del quirófano. Al finalizar la consulta, se leyeron otros dos libros: el primero, titulado “¿Ya tienes una marquita?”, de 20 páginas, que narraba la trayectoria de un niño con fisura orofacial durante su rehabilitación^(10)^. El segundo libro se titulaba “Cuando me siento feliz”, de 15 páginas, y trataba sobre el sentimiento de felicidad^(11)^. Con el consentimiento de los tutores, se grabaron la voz y la imagen del niño durante toda la consulta.

### Análisis y Procesamiento de los Datos

Los datos se analizaron de manera descriptiva, utilizando la grabación de la consulta, las anotaciones de enfermería y las fotos que se tomaron.

### Aspectos Éticos

La investigación respetó todas las directrices éticas. Fue aprobada por el Comité de Ética en Investigación con Seres Humanos de la universidad signataria, mediante el Dictamen n^.o^ 7.041.250/2024 (Certificado de Presentación para Evaluación Ética n^.o^ 81990024.0.0000.0104). Además, se aplicaron el Formulario de Consentimiento Libre e Informado y el Formulario de Asentimiento Libre e Informado, ambos en dos ejemplares. Se obtuvo el consentimiento de los tutores legales y el asentimiento de la niña para la grabación de su voz e imagen, así como para el uso del dibujo realizado durante la sesión, preservando el anonimato y la confidencialidad de la información.

## RESULTADOS

### Evaluación de Enfermería

La niña, de 8 años de edad, fue diagnosticada con fisura completa post foramen. Se encuentra en seguimiento fonoaudiológico y recibe apoyo pedagógico en una asociación de apoyo y, a petición de la madre, retomó el seguimiento psicológico. Se sometió a su primera cirugía de palatoplastia en 2018, a los dos años de edad, lo que provocó una fístula que aún no se ha corregido.

La niña tenía programada una cirugía de corrección de la fístula para 2022, momento en el que presentó síntomas de ansiedad y el procedimiento no se llevó a cabo, a pesar de recibir atención psicológica. La cirugía se programó nuevamente para junio de 2025, y la niña volvió a presentar síntomas de ansiedad y miedo a la bata blanca.

Inicialmente, se llevó a cabo una atención de enfermería individual con la madre de la niña, quien relató haber pasado por una experiencia traumática durante la primera intervención quirúrgica de su hija, debido al miedo y la ansiedad causados por la demora en el procedimiento. Al enterarse de que su hija se sometería a otra cirugía, la madre volvió a sentirse aprensiva. Por ello, se le brindó orientación sobre las normas y rutinas hospitalarias, sobre el procedimiento y sobre la importancia de mantener la calma, ya que los cuidadores son el punto de referencia y seguridad para los niños, y muchas veces su comportamiento es un reflejo del de los padres^(12)^.

Se llevó a cabo una sesión individual con la niña, utilizando estrategias lúdicas para facilitar su expresión y comunicación verbal y no verbal. Inicialmente, se leyó un libro sobre el miedo^(9)^. En ese momento, la niña ya expresó sus sentimientos al respecto, comentando: “*Me pareció chido* (la historia). *Hay que enfrentar el miedo, y yo tengo miedo de la cirugía*”.

A continuación, para comprender realmente cuál era el miedo de la niña respecto al procedimiento, se le pidió que hiciera un dibujo a partir de la pregunta: “¿Cuál es tu miedo al someterte a la cirugía?”. Al terminar, se le pidió que explicara su dibujo, y ella dijo: “*Dibujé a mí y a la enfermera. Ella está soltando un poco de humo. Tengo miedo de desmayarme y no volver a despertarme nunca más; no sé por qué, pero tengo miedo, porque entonces no voy a vivir. Solo tengo miedo de entrar al quirófano. Ya fui una vez, pero no lo recuerdo, porque era un bebé*”. El dibujo se muestra en la Figura 2.

**Figura 2:** Dibujo realizado por la niña sobre el miedo a la cirugía.

**Fuente:** Datos de la investigación.

La niña interactuó de manera activa durante toda la sesión, expresando todas sus dudas y preocupaciones. Al demostrar, con los juguetes, que sería necesaria una punción venosa, el niño dijo: “*¿Me van a pinchar mientras estoy durmiendo? ¿La van a dejar ahí* (la aguja) *hasta que termine la cirugía?*”. La niña también reveló su ansiedad relacionada con la anestesia: “*Me van a dar el humito para que me duerma* (en el quirófano). *Cuando me pongan el humito, ¿me voy a dormir de inmediato? ¿Me va a tomar tiempo dormirme?*”.

Otra curiosidad que expresó fue saber si habría más niños en el hospital o si estaría sola: “*¿Donde yo voya estar, también habrá más niños?*”. Además, la muñeca se asoció con la seguridad de la niña durante el procedimiento, y ella preguntó: “*¿Ella* (la muñeca) *podrá quedarse en el quirófano conmigo?*”.

Al final de la sesión, la niña comprendió el objetivo de la intervención quirúrgica para su rehabilitación: “*No voy a poder ver* (la sutura). *Solo lo voy a poder notar por la voz, ¿no?* (cambio y mejora en la voz después de la cirugía)”. Además, expresó su opinión sobre la sesión de JT: *”A* mí me pareció (la sesión de JTI) *genial, ya no tengo miedo. Creo que mi mamá me va a tranquilizar* (el día de la cirugía)”.

Al final de la sesión, se leyó un libro que cuenta la historia de un niño con fisura orofacial y su rutina de tratamiento^(10)^. Posteriormente, también se leyó un libro sobre la felicidad^(11)^. Al terminar, la niña expresó sentimientos positivos respecto a la sesión: “(cuando me operen) *me voy a sentir feliz de haberlo logrado*”. Además, se dio cuenta de que la sesión de terapia conductual fue importante para tranquilizarla: “*Tengo menos miedo, me he tranquilizado*”.

### Diagnósticos de Enfermería

A partir del JTI, al identificar las necesidades y los problemas de la niña, se establecieron cuatro diagnósticos de enfermería, de acuerdo con la NANDA-*International*, tal como se muestra en la Cuadro 1 ^(13)^.

**Cuadro 1:** Diagnósticos de enfermería – Maringá, PR, Brasil, 2025.

Ámbito	Diagnóstico de enfermería	Características definitorias	Factores relacionados
Ámbito 9 – Afrontamiento/tolerancia al estrés	Ansiedad excesiva	Inseguridad, nerviosismo y tensión	Situación desconocida (cirugía), asociada a procedimientos invasivos
Ámbito 9 – Afrontamiento/tolerancia al estrés	Miedo excesivo	Aprehensión, disminución de la confianza en uno mismo y nerviosismo	Situación desconocida (cirugía) y población de riesgo infantil, asociada a procedimientos invasivos
Ámbito 9 – Afrontamiento/tolerancia al estrés	Mayor disposición para afrontar el estrés	Deseo de mejorar la gestión del estrés	
Ámbito 9 – Afrontamiento/tolerancia al estrés	Mayor disposición a la resiliencia	Deseo de mejorar la perspectiva positiva, la sensación de control y las habilidades de afrontamiento	

### Aplicación de la Enfermería

De este modo, se establecieron las siguientes posibles intervenciones de enfermería, de acuerdo con la Clasificación de Intervenciones de Enfermería^(14)^, que se recogen en la Cuadro 2.

**Cuadro 2:** Prescripciones de enfermería – Maringá, PR, Brasil, 2025.

Diagnóstico de enfermería	Prescripciones de enfermería
Ansiedad excesiva	– Explicar al niño el procedimiento y las rutinas hospitalarias. – Informar sobre los equipos que se van a utilizar. – Utilizar juguetes para simular los procedimientos. – Utilizar el juguete terapéutico instructivo para la preparación preoperatoria. – Evaluar continuamente los signos de ansiedad antes y después de las intervenciones.
Miedo excesivo	– Fomentar la expresión de los sentimientos y percepciones sobre el miedo. – Validar los sentimientos del niño y asegurarle que es normal sentir miedo. – Proporcionar un entorno seguro y acogedor. – Utilizar el juguete terapéutico para expresar los sentimientos relacionados con la intervención quirúrgica. – Supervisar y reevaluar la intensidad del miedo a lo largo del seguimiento.
Mayor disposición a afrontar la situación	– Explorar los sentimientos y las preocupaciones relacionados con la situación actual. – Realizar un seguimiento de la evolución de las estrategias de afrontamiento adoptadas por el niño.
Mayor capacidad de resiliencia	– Fomentar la esperanza y el optimismo mediante ejemplos positivos. – Evaluar el mantenimiento de actitudes positivas ante la intervención quirúrgica.

## DISCUSIÓN

El caso en cuestión presenta manifestaciones de ansiedad y miedo asociadas al procedimiento quirúrgico, aspectos que suelen estar relacionados con experiencias comunes en la vida de los niños con fisuras orofaciales. Estos niños requieren diversas intervenciones quirúrgicas a lo largo de su crecimiento y desarrollo, lo que puede despertar sensaciones emocionales negativas, como miedo, ansiedad y pánico, especialmente si ya cuentan con un contexto y experiencias traumáticas previas^(15)^.

Un estudio realizado en un hospital de Brasil identificó que la incorporación del JT en la atención médica trajo resultados positivos para la práctica de los profesionales, para la institución y para los familiares de los niños hospitalizados en una Unidad de Terapia Intensiva, lo que indica que el uso del juguete es transformador. Para implementar el juguete en la atención, se identificaron las barreras para su uso, se planificaron mejoras, se definieron metas, se designó a un enfermero líder, se establecieron alianzas entre profesionales, se promovieron capacitaciones para el equipo y se creó un grupo de referencia para el JT y un protocolo institucional^(16)^.

Un estudio cuasi-experimental realizado en Brasil, al considerar la comodidad de los niños, verificó que el uso del JTI favoreció su comportamiento colaborativo y fue responsable de reducir en un 43 % el nerviosismo, en un 51 % el movimiento y la falta de protesta, y de aumentar en un 27 % el comportamiento colaborativo en niños sometidos a cateterización intravenosa periférica^(7)^.

Los hallazgos de este estudio concuerdan con la literatura, ya que evidencian la importancia del JTI en la preparación del niño, aumentan la confianza y reducen el miedo al procedimiento. Además, otra investigación realizada en Turquía utilizó títeres de mano con niños en edad preescolar durante el procedimiento de recolección de sangre, y los resultados determinaron que la estrategia redujo el miedo y el dolor de los niños durante el procedimiento^(17)^.

En este sentido, se considera necesario incorporar estrategias que promuevan la comodidad, la confianza y la seguridad del niño, tomando en cuenta las necesidades especiales de cada grupo de edad. De esta manera, el JT puede incorporarse en todos los contextos de atención de la salud infantil, con la participación de todo el equipo multiprofesional^(18)^.

De este modo, el equipo de enfermería que trabaja en pediatría cuenta con el respaldo de la Resolución n.º 546/2017 del Consejo Federal de Enfermería para el uso de juguetes y la terapia de juego en la atención a los niños y a sus familiares hospitalizados. De acuerdo con la resolución, esta técnica debe abarcar las etapas del proceso de enfermería y debe constar en el expediente médico^(19)^.

Además, se pudieron identificar diagnósticos de enfermería que reflejaban las necesidades del niño ante el procedimiento quirúrgico. Los diagnósticos “Ansiedad excesiva” y “Miedo excesivo” revelan los sentimientos de inseguridad y miedo a la anestesia^(13)^. La ansiedad y el miedo, con frecuencia, se evalúan de manera subjetiva en pacientes pediátricos quirúrgicos. Un estudio realizado con enfermeros brasileños identificó que, para estos profesionales, brindar información confiable al paciente durante la preparación preoperatoria es crucial para reducir el miedo y la ansiedad durante el tiempo de permanencia en el quirófano^(20)^.

Por su parte, los diagnósticos “Disposición mejorada para el afrontamiento” y “Disposición mejorada para la resiliencia” evidencian que el niño es capaz de desarrollar estrategias de afrontamiento cuando se le atiende de manera lúdica y sensible^(13)^. El uso de recursos lúdicos le permite al niño expresar emociones y sentirse más seguro, lo que contribuye a aumentar el autocontrol y la confianza, aspectos esenciales para la capacidad de afrontamiento y la resiliencia infantil^(21)^.

El estudio presenta limitaciones relacionadas con la realización de una sola sesión para preparar al niño para el procedimiento quirúrgico y la ausencia de seguimiento posoperatorio, lo que permitiría comprender mejor los beneficios de la intervención a largo plazo. Además, al tratarse de un estudio de caso, realizado en una sola asociación de apoyo, existe una limitación en cuanto a la generalización de los hallazgos. Sin embargo, este hecho se justifica debido a la rutina de atención de la asociación y a la falta de asistencia del niño y de sus responsables a las citas programadas.

A pesar de ello, el estudio ofrece aplicaciones valiosas para la práctica de la enfermería, lo que respalda la incorporación de el JT en las consultas de enfermería, lo cual fortalece la atención pediátrica. Además, la investigación ofrece aportaciones y recursos que pueden implementarse en otros contextos pediátricos que tengan como objetivo preparar al niño y reducir la ansiedad en el ámbito de la salud.

## CONCLUSIÓN

El estudio evidenció que incorporar el JTI en la consulta de enfermería fue una estrategia eficaz en la preparación quirúrgica de niños con fisuras orofaciales. El caso de estudio demostró que la intervención permitió que el niño expresara sus sentimientos de miedo y ansiedad, lo que facilitó la comprensión del procedimiento.

El estudio refuerza la necesidad de implementar el JTI como una estrategia sistematizada en la práctica de la enfermería pediátrica, especialmente en la preparación para procedimientos invasivos, lo que contribuye a la humanización de la atención.

## Data Availability

Todo el conjunto de datos que apoya los resultados de este estudio fue publicado en el propio artículo.
